# Increasing the Hydration Activity of Tricalcium Silicate by Adding Microdispersed Ettringite as a Nucleating Agent

**DOI:** 10.3390/ma16227078

**Published:** 2023-11-08

**Authors:** Yurii R. Krivoborodov, Svetlana V. Samchenko, Andrey V. Korshunov, Irina V. Kozlova, Dmitrii G. Alpacky

**Affiliations:** Department of Building Materials, Moscow State University of Civil Engineering, 26, Yaroslavskoye Shosse, 129337 Moscow, Russia; ykriv@mail.ru (Y.R.K.); samchenko@list.ru (S.V.S.); ivkozlova@mail.ru (I.V.K.); alpacky@mail.ru (D.G.A.)

**Keywords:** tricalcium silicate, hydration, microdispersed ettringite, nucleation sites, kinetics of setting, bending strength

## Abstract

Tricalcium silicate (C_3_S) as a binder material has a decisive influence on the processes of hardening and strength gain of cements and concretes. One of the promising directions is the introduction of dispersed additives into cement mixtures, which allow micro-level control of the composition of hydration products and change the dynamics of the structure formation of cement stone. In this paper, the effect of a microdisperse ettringite additive on the kinetics of the hydration and hardening process of tricalcium silicate was studied. It was shown that ettringite crystals selectively adsorb Ca^2+^ and OH^−^ ions from a saturated solution of calcium hydroxide, which contributes to the formation of hydrosilicate nuclei on their surface during cement hydration. Hydration of C_3_S in the presence of ettringite proceeds more intensively; the addition of ettringite contributes to an increase in the content of calcium hydrosilicates in hydration products at the initial stage of the process. Addition of 10 wt.% ettringite to C_3_S reduces the induction period of the beginning of the main phase of heat release by around two times and increases the amount of heat released on the 1st day of hydration by 15% compared to the control sample. According to electron microscopy data, it was found that during the first hours of hydration of modified C_3_S, a significant number of nuclei of fibrous particles of calcium hydrosilicates with sizes of 0.2–2 microns were formed on the surface of ettringite crystals. According to the results of kinetic modeling of the setting process of cement pastes using the Avrami–Erofeyev model, it was shown that in the presence of the addition of microcrystals of ettringite, the setting rate is characterized by a slowdown in nucleation, whereas for a sample without an additive, this process proceeds with an acceleration of the formation of solid-phase nuclei. Based on the comparison of kinetic results and mechanical measurements, it is concluded that needle crystals of ettringite during C_3_S hydration and cement stone hardening are preformed centers for the growth of hydrosilicate nuclei, and they also act as a reinforcing filler, increasing the bending strength of modified samples. The results of the work can be used in practice in the development of methods for controlling the processes of hydration and hardening of cements, as well as for controllable structure formation of cement stone which is important in particular for 3D printing of building products and constructions.

## 1. Introduction

A significant number of scientific research papers have been devoted to the theory of hydration and hardening of binders [1,2,3,4]. However, there is still no common approach to the processes occurring during the interaction of cement with water, leading to the formation of strong structures. Similarly, there is an open question concerning cement components or hydration products which determine the initial and final strength of cement stone [5,6,7].

Tricalcium silicate (C_3_S) is the most important component of Portland cement, because it mainly contributes to the hardening and strength gain, as well as the durability of cements, mortars and concretes [8,9,10,11,12].

C_3_S dissolves rapidly in water, releasing calcium, silicon and hydroxide ions [1]. The concentration of the solution increases up to a critical saturation with respect to C-S-H, causing its precipitation at a significantly lower Ca/Si ratio than in C_3_S, which leads to a decrease in silica concentration and an increase in calcium in the pore solution [1,13,14,15]. The issue of the induction period of C_3_S hydration, as well as the beginning of the rapid acceleration period following the dormant period, is widely discussed in the scientific literature [8,15,16,17].

Much attention is paid to the strength gain acceleration of cement composites modified with nano additives [18,19,20,21,22,23,24,25,26]. The use of nanoscale particles in the designing of building materials allows control of the processes of cement hardening at micro-level and specifically control of the composition of hydration phases [19]. The efficiency of using nanoscale particles for structuring cement pastes consists in (1) ensuring the cement paste mobility at the initial period of hydration as well as the plasticizing effect without blocking the surface of cement particles with surfactants, (2) making it possible to obtain a dense and durable cement matrix with improved mechanical characteristics [18,19,20,21,22,23,24].

It is believed that the loss of mobility of cement paste and an increase in early strength occurs due to the formation of calcium hydrosulfoaluminates. The formation of calcium hydroaluminates and hydrosulfoaluminates, mainly ettringite, in the early hydration period is a factor leading to an increase in the strength of cement stone, since the ettringite crystals reinforce the cement stone [27,28,29,30]. To increase the amount of ettringite or its analogues, calcium sulfoaluminates are added to cement composite [31,32,33,34,35], or the amount of gypsum additive is increased [31,36,37,38].

A number of publications [1,2,3,9,39] demonstrate that the rate of structure formation is related to the amount of calcium hydrosilicates formed at an early stage of hardening, especially they improve the durability of cement stone and its strength at 28 days.

There are works devoted to the application of various crystalline additive analogues of cement stone hardening products as intensifiers of the hardening process [40,41]. These crystalline additives mainly affect the hydration rate of the silicate component of cement. The effect of crystalline calcium hydrosilicate additives on cement hydration [42,43,44,45,46,47,48,49], as well as additives of calcium hydrosulfoaluminates [50], has been well studied. However, there are still questions about the effect of these crystalline additives on the hydration of the main cement minerals.

Cement hydration is often modeled as the process of nucleation and growth of crystal hydrates during the interaction of cement minerals with water [26,50,51,52,53,54]. The products of early hydration are formed continuously [27,28,30,44]; therefore, this process makes a significant contribution to the thixotropic structure formation of cement pastes [54], as well as to the rheological characteristics of cement pastes [55,56,57,58].

The introduction of crystalline additives is a useful way to study the mechanisms of early hydration of both individual cement minerals and their combination. This is essential because the accelerating properties of crystalline additives depend on nucleation and growth, which are the rate-limiting stages in the hydration process. The presence of crystalline additives has an insignificant or no effect on the composition of the pore solution. If the rate-limiting stage of cement minerals hydration is the rate of their dissolution, then such additives should have little or no effect on the kinetics. However, if the rate-limiting reactions are nucleation and growth, then the effect of crystalline additives as crystallization centers will lead to a significant acceleration of the hydration process of cement hardening.

Such crystalline additives are quite difficult for manufacturing and therefore could not be widely used in industry. In this regard, much attention is currently being paid to Supplementary Cementitious Materials (SCM’s) [59,60], the mineralogical composition of which is represented by calcium aluminosilicates. Their behavior in the cement compositions is due to the interaction of calcium hydroxide formed during hydration of alite, the main mineral of Portland cement, with the formation of calcium hydroaluminosilicates and low basic calcium hydrosilicates [59,60,61,62,63].

A quite simple and interesting technological decision, but little studied, is the method of introducing a part of the cement into the mixing water [56,64,65]. In this case, the additive particles act as crystallization centers and accelerate the crystallization of hydrate compounds, while the mixing water is enriched with compounds for the synthesis of these hydrate phases.

A better understanding of the cement mineral hydration mechanisms in the presence of crystalline additives is necessary to develop new methods for activating and optimizing the properties of cement pastes and concretes in general.

The purpose of this work was to conduct comprehensive studies of the kinetics of the hydration and hardening process of C_3_S, the main cement mineral responsible for strength gain, with a synthesized microdisperse additive of ettringite.

## 2. Materials and Methods

### 2.1. Materials Used in the Study

#### 2.1.1. Synthesis of Tricalcium Silicate

C_3_S was synthesized by calcination of stoichiometric mixtures of CaO and SiO_2_ oxides (Sigma-Aldrich, St. Louis, MO, USA, purity ≥ 99.99%) without additional purification. Powdered oxides were thoroughly mixed until homogeneous mixtures were obtained, pressed into pellets at a pressure of 0.7 MPa, placed into Pt crucibles, heated to 1600 °C and calcined under isothermal exposure conditions for 8 h. After isothermal exposure, the furnace was turned off, and the crucibles with samples were taken out from the furnace and cooled to room temperature. The resulting sintered samples were ground in an agate mortar and sieved through a sieve with a cell size of 63 μm.

**Figure 1:**
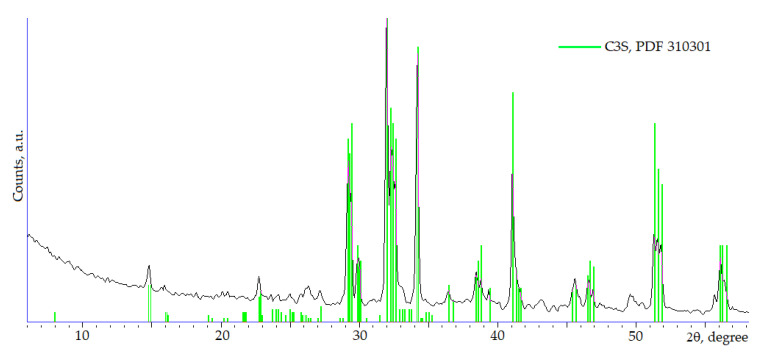
XRD pattern of C_3_S.

The chemical composition of C_3_S was determined in accordance with the ASTM C114-11 standard [66]. To determine the SiO_2_ content, a sample of material weighing 0.5 g was placed in a platinum crucible, 10 drops of concentrated sulfuric and hydrofluoric acids were added, and the resulting mixture was evaporated under low heating to a moist state. Next, 5 mL hydrofluoric acid was added to the residue again and evaporated to a dry state. Then, the resulting residue was calcined in a muffle furnace at 900–1000 °C for 15 min; the crucible was then cooled in a desiccator and weighed. The whole procedure was repeated 2–3 times until a constant mass of the crucible with a dry residue was reached. The mass fraction of SiO_2_ in the analyzed sample was determined by the loss of mass after calcination. The content of calcium, aluminum, and iron oxides was determined in the residue by fusing it with a small amount of a mixture of sodium carbonate and borax (2:1) at 900–950 °C for 5 min, followed by dissolution of the melt in hydrochloric acid (1:3), precipitation of aluminum and iron oxides with ammonia and subsequent precipitation of calcium from the filtrate with ammonium oxalate. Iron and aluminum oxides in the sediment were determined by titration with 0.05 M Trilon B in the presence of sulfosalicylic acid and reverse titration with ferric chloride, respectively. Calculations of the oxide content based on the results of the analyses were carried out in accordance with ASTM C114-11; the results are shown in Table 1. The purity of the obtained mineral was examined using X-ray diffraction (SHIMADZU XRD 6000, Shimadzu Corp., Kyoto, Japan) (Figure 1).

#### 2.1.2. Synthesis of Ettringite

Ettringite synthesis was carried out by precipitation from solution, which is considered the most optimal way because of the simplicity of the technique and low energy consumption. To obtain ettringite in the laboratory, the following materials were used: sugar; Ca(OH)_2_; Al_2_(SO_4_)_3_·18H_2_O and sodium hydroxide. The method of obtaining the additive included the following steps (all operations were performed at room temperature). Solution A: 20 g of sugar and 6 g of calcium hydroxide were dissolved in 180 mL of distilled water. Solution B: aluminum sulfate hydrate Al_2_(SO_4_)_3_·18H_2_O weighing 17.98 g was dissolved in 200 mL of water, and the pH of the solution was adjusted to 12.0 ± 0.5 by adding solid sodium hydroxide in small portions while stirring. Both solutions obtained should be transparent without suspension or sediment; if necessary, the solutions should be filtered. Next, solution B was added drop by drop to solution A with constant intensive stirring. The chemical reaction between the reagents taken is as follows:

6Ca(OH)_2_ + 2Na[Al(OH)_4_] + 3Na_2_SO_4_ + 26H_2_O = Ca_6_Al_2_(SO_4_)_3_(OH)_12_·26H_2_O↓ + 8NaOH

The resulting ettringite suspension was filtered through a paper filter, washed with a fivefold volume of distilled water and dried at room temperature. The dried product was ground in an agate mortar and stored in a closed container. In order to avoid contamination of ettringite with calcium carbonate, all solutions were prepared using pre-boiled distilled water. Synthesis and drying of ettringite was carried out in a nitrogen-filled box (Nitrogen glove box, Cleatech LLC, Orange, CA, USA).

#### 2.1.3. Preparation of Model Mixtures

To conduct comprehensive studies of the kinetics of the hydration and hardening process of C_3_S with a synthesized microdisperse additive of ettringite, a control and model mixtures were prepared. The model mixtures contained finely ground synthesized C_3_S and 10 wt.% of synthesized ettringite. This amount of the additive was chosen for the following reasons. Firstly, the upper value of the additive equal to 10 wt.% is the maximum if its particles are considered as crystallization centers, and secondly, an increased level of the additive will have a more noticeable effect on the hydration process, phase composition, structure and hydration degree. Introduction of the additive of less than 10 wt.% slightly affects the results of mechanical measurements and gives less pronounced changes in microscopic, thermal and other measurements. In this regard, for a more detailed consideration of the processes occurring during C_3_S hydration with the addition of ettringite, the data for cement pastes of standard consistency based on C_3_S with the addition of 10 wt.% of ettringite are presented in the paper.

Precisely measured quantities of raw materials were carefully crushed using an agate mortar. After grinding, the mixture was sieved through a sieve with a cell size of 63 microns. Required amounts of mixture components were placed into the mixing bowl. At first, cement was placed into the bowl, and the mixing water was poured into the center of the cement. Reference samples did not contain ettringite. The additive was introduced into the cement paste together with the mixing water.

Distilled water was used for mixing. The standard consistency of the C_3_S paste was 0.42, and the standard consistency of the paste with the addition of ettringite was slightly higher, at 0.45, which was probably due to a significant acceleration of crystallization processes in the presence of an additive.

After water was added into the cement, the mixture was thoroughly homogenized for 5 min and then cast into the 1 × 1 × 3 cm-sized molds. To prevent the samples from sticking to the molds during their subsequent extraction, the molds were coated with oil, and excess oil was removed with a dense, dry cloth. After that, the molds with the mixture were installed on a vibrating platform and vibrated for 2 min to evenly distribute the composition, as well as to remove the remaining air bubbles from the mixture. Then, the molds were placed into a humid environment (22 ± 1 °C; 98–100% relative humidity) until hardened.

### 2.2. Research Methods

#### 2.2.1. Thermal Analysis

The SDT Q600 (TA Instruments, New Castle, DE, USA) thermal analyzer was used to perform thermal analysis of the samples. Mass changes of the sample (thermal gravimetric analysis) and processes accompanied by the release or absorption of heat (differential scanning calorimetry/differential thermal analysis) were evaluated simultaneously under the conditions of a heating rate of 10 K/min and an air flow of 100 mL/min.

The amount of dry residue from calcium silicates, solid residue from the additive and the amount of water introduced into the system with the additive were determined by the mass loss of the samples from C_3_S with the additive when heated up to 1000 °C according to the following formulas:

1. The amounts of dry residue in the analyzed mixture were determined by

G_dry_ = G − ∆G^20–1000^ (1)

where G is the total mass of the sample for the analysis;

G^20–1000^ is the mass loss of the sample when heated from 20 to 1000 °C, while the total amount of dry residue can be represented as follows:

(2) $$G_{dry}=G_{{C_{3}S}_{unhydrous}}+G_{{C_{3}S}_{hydrate}}+G_{additive}$$

where G_C3Sanhydrous_ and G_C3Shydrated_ are the amounts of dry residue, respectively, from the non-hydrated and hydrated portion of calcium silicates, and G_additive_ is the amount of dry residue that is introduced with the additive.

Then:

(3) $$G_{{C_{3}S}_{\mathrm{unhydrous}}}+G_{{C_{3}S}_{\mathrm{hydrated}}}=G_{\mathrm{dry}}-G_{\mathrm{additive}}$$

2. The amount of dry residue was determined, which was introduced with the addition of ettringite (C_3_A∙3CaSO_4_∙32H_2_O).

If we assume that *m* is the mass (wt.%) of the additive in the mixture, and (100–*m*) is the mass (wt.%) of C_3_S in the dry residue from calcium silicates, then the amount of dry residue from calcium silicates, the amount of solid residue introduced with the additive and the amount of water introduced into the system with the additive can be expressed in weight parts and the amount of dry residue from hydrated and non-hydrated C_3_S can be calculated using the (1) and (2) formulas and the data in Table 2.

#### 2.2.2. X-ray Diffraction (XRD) Data

XRD patterns were obtained using a SHIMADZU XRD 6000 diffractometer (SHIMADZU XRD 6000, Shimadzu Corp., Kyoto, Japan) with a Cu-anode (λ_Ka1_ = 1.54056 Å; 40 mA and 40 kV). X-ray diffractograms were taken at a 5 to 70° range of 2θ with a scanning step of 0.02°. The identification of starting minerals was carried out according to the ICDD PDF-2 database. Quantitative determination of hydration products using X-ray diffraction is not possible, especially within the early age, due to roentgen-amorphousness of the resulting hydrosilicates. In this regard, the relative content of phases with the highest degree of crystallinity (C_3_S, ettringite, portlandite) was determined qualitatively by the most pronounced reflexes at the following angles 2θ: C_3_S 32°; ettringite 9°; calcium hydroxide CH 18°.

#### 2.2.3. Infrared Microscopy

The method of IR spectroscopy was used to study the chemical composition of the obtained samples. For this purpose, the IR spectra of the material were taken from the surface of the samples in the region of 500 ÷ 4000 cm^−1^ in KBr using a Nicolet 4700 IR Fourier spectrometer (Thermo Fisher Scientific, Waltham, MA, USA).

#### 2.2.4. Scanning Electron Microscopy (SEM)

The microstructure of the hydrated samples was examined using a Hitachi S-450 scanning microscope (Hitachi High-Tech Corp., Tokyo, Japan) with a resolution of 150 Å. Samples for scanning microscopy were prepared immediately before the study. The fracture surface of the samples, on which a conductive layer of gold was sprayed, was analyzed. Spraying was carried out on a special device; the coatings were uniform, and their thickness did not exceed 30–40 Å.

#### 2.2.5. Determination of ζ-Potential

The ability to absorb certain ions from the solution is determined by the properties of the surface of the additive. One of the characteristics of the interface is the electrokinetic potential (ζ-potential). The value of the ζ-potential was measured using a Zetasizer Nano ZS analyzer (Malvern Instruments, Malvern, UK) for ettringite after 1 h after holding it in distilled water.

#### 2.2.6. Adsorption

The adsorption capacity of the synthesized ettringite crystals in relation to a saturated solution of calcium hydroxide was determined by measuring the concentration of Ca^2+^ and OH^−^ ions in a saturated solution of calcium hydroxide. To conduct this experiment, 1 g ettringite was placed into a graduated flask and 100 mL of saturated Ca(OH)_2_ solution was added. At certain time intervals (15 min, 30 min, 1 h, 6 h, 12 h, 18 h, and 24 h), a portion of the solution was taken and the concentrations of Ca^2+^ and OH^−^ ions were determined.

#### 2.2.7. Chemical Analysis

The content of Ca^+2^ ions in the liquid phase was determined by titration with a Trilon B solution in the presence of the acidic chrome blue indicator with the addition of a 20% potassium hydroxide solution until the color changed from pink to lilac-blue.

To determine the concentration of OH^−^ ions, a standard method of titration with hydrochloric acid solution in the presence of a methyl orange indicator was used until the color transitioned from yellow to pink. The dynamics of pH change in the liquid phase was determined using a pH meter, while a glass electrode was used as a selective electrode, and a silver chloride electrode was used as a reference electrode.

#### 2.2.8. Determination of the Hydration Degree

The hydration degree of the mineral alite was determined by quantitative X-ray analysis by the intensity of the analytical peak with d = 0.1761 nm (2θ = 51.8°, Figure 1). The calculation was carried out according to the formula

(4) $$\alpha (\%)=100-\frac{I_{t}}{I_{0}}\cdot 100$$

where α is the hydration degree and *I_t_* and *I*_0_ the peak intensity of the hydrated and initial samples, respectively.

#### 2.2.9. Calorimetry

The kinetics of heat release during the hydration process in a cement paste with W/C ratio of 0.5 was studied using an isothermal calorimeter TAM AIR (TA Instruments). Based on the heat-release results, differential and integral heat-release curves were constructed.

#### 2.2.10. Determination of the Strength of Model Mixtures

To determine the strength of samples modified with a microdisperse additive of ettringite, 1 × 1 × 3 cm-sized beam samples were formed from cement paste with a W/C ratio corresponding to standard consistency. The samples were compacted on a vibrating platform with a frequency and amplitude of 3000 vibrations per minute and 0.35 mm, respectively, for 120 s. The hardening process was performed in a humid environment at 22 °C.

The bending strength of the samples was determined on a PM–A-70AB press with the registration of the load-bending diagram with a loading rate of 0.4 mm/min; the compressive strength for 5 × 5 × 5 cm-sized samples was determined using a hydraulic press CONTROLS MCC8 50-C8422 (Controls s.r.l., Milan, Italy) with a loading rate of 2 MPa/s. Mechanical tests for each series of samples were repeated 6 times after 1, 3, 7, and 28 days; the average values were calculated from the data obtained, and the standard deviation was determined.

#### 2.2.11. Determination of the Dynamics of the Solid-Phase Nucleation during the Setting of the Mixture

The standard consistency and setting time of the cement pastes were determined using a Vicat apparatus (Gilson Co., Lewis Center, OH, USA).

The dynamics of the solid-phase grain nucleation was evaluated using the Avrami–Erofeyev model (5) [67], which is most often used to describe the kinetics of topochemical and solid-phase processes, the rate of which is determined by the formation and growth of nuclei of solid phases:

(5) $$\alpha =1-exp(-K\times \tau^{n})$$

where α = h/h_max_ is the relative distance to which the Vicat apparatus needle penetrates the cement paste;

*K* is the empirical constant of the grain growth rate;
τ is time;
*n* is a parameter of the grain growth dynamics.
Values of the parameter *n*:
*n* > 4: increasing rate of nucleation;
*n* = 4: constant rate of nucleation;
*n* = 3–4: decreasing rate of nucleation.

## 3. Results and Discussion

Calcium silicates are the main component of cement; the products of their hydration determine both the early and 28-days-of-age strength of cement stone [10]. The early strength is mainly provided by the mineral of cement clinker alite C_3_S. Belite C_2_S is hydrated at a lower rate, which contributes to the formation of a strong structure at the later hardening period [3,7].

Currently, there is no common opinion on which stage is the limiting in the process of hardening of these minerals immediately after mixing with water—the stage of dissolution or the nucleation of a new phase [43]. It is considered that some additives act as crystallization centers, accelerating the hydration process and hardening of mineral binders. They affect the crystallization rate of the main structure-forming phase or the conditions for films crystallization [24]. However, there is practically no direct evidence of this in the literature. The mechanism of action of ettringite additive and other calcium hydroaluminates is also insufficiently studied.

### 3.1. Adsorption Capacity of Synthesized Ettringite Crystals

**Figure 2:**
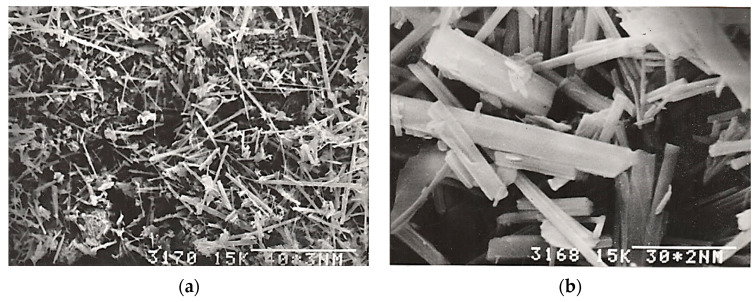
Crystals of the synthesized ettringite: (**a**) the largest needle-shaped crystals up to 50 microns long and up to 3 microns in diameter; (**b**) small fraction crystals up to 5 microns long and up to 0.5 microns in diameter.

The obtained ettringite consisted of needle crystals from 5–50 μm long and from 0.5–3 μm in diameter. The length/diameter ratio was 10–15 (Figure 2).

**Figure 3:**
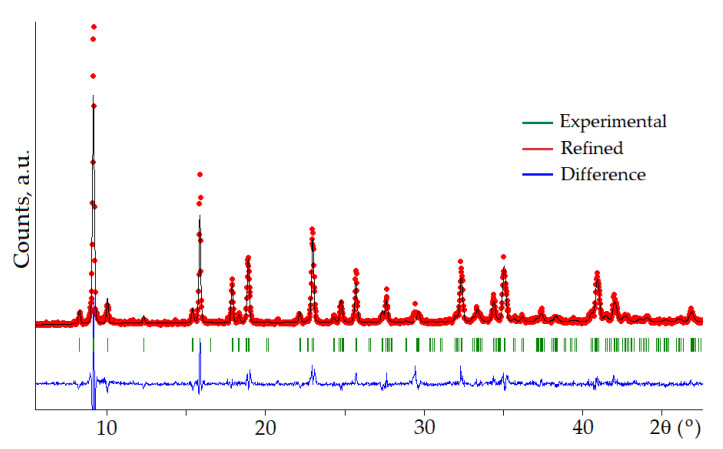
Results of the Rietveld refinement of ettringite diffraction pattern (χ^2^ = 2.2).

**Figure 4:**
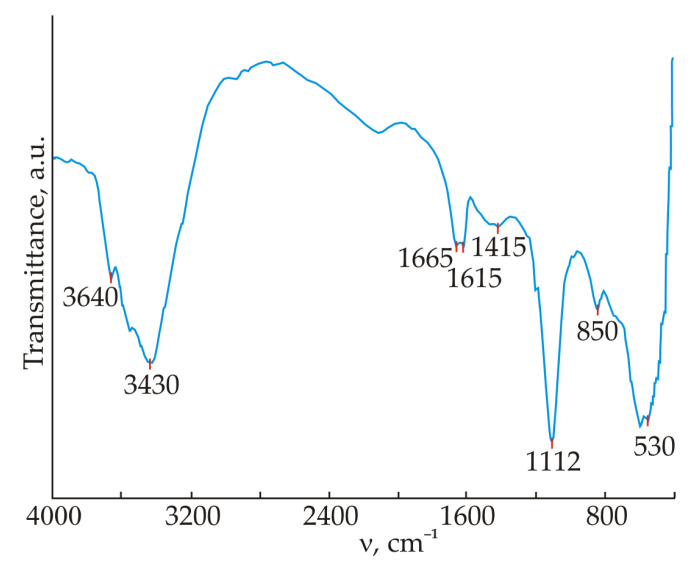
IR spectrum of ettringite.

The purity of the obtained ettringite was investigated by XRD analysis (Figure 3) and IR-spectroscopy (Figure 4). The XRD pattern showed that the obtained product contains mainly ettringite. Absorption bands in the region of 3640 cm^−1^ (O-H bond vibrations), 3430 cm^−1^ and 1665 cm^−1^ (stretching and bending vibrations of H_2_O bonds), 1112 cm^−1^ (SO_4_^2−^ bond vibrations), 850 cm^−1^ (Al(OH)_6_ bond vibrations were detected in the IR spectrum, which is in good agreement with literature data [68].

**Figure 5:**
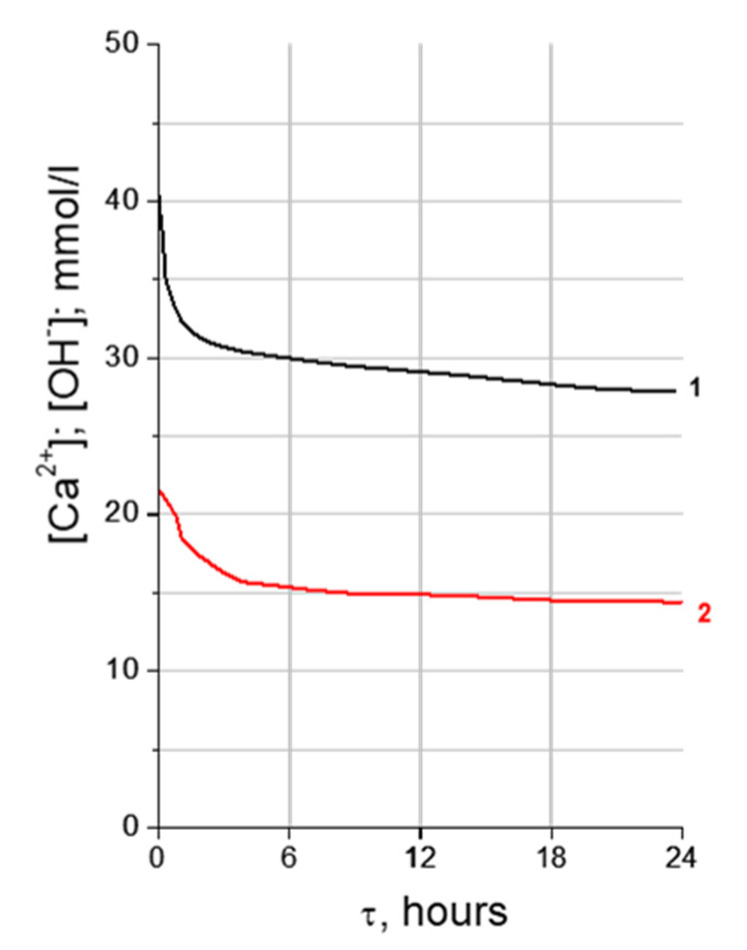
Change in Ca^2+^ (1) and OH^−^ (2) ion concentration in Ca(OH)_2_ saturated solutions with ettringite additive.

The process of hydration product formation can be considered as a process of crystallization from supersaturated solutions. Calcium and hydroxide ions are involved in almost all reactions that occur during the hydration of cement. The change in the concentration of calcium and hydroxide ions characterizes the rate of the hydration reaction and, mainly, the yield of the final product. The ability of active mineral additives and calcium hydrosilicates to absorb calcium hydroxide from its saturated solutions is well known [59]. There is practically no information about the ability of ettringite to absorb calcium hydroxide from its saturated solutions. Therefore, in this article the authors investigated the adsorption capacity of synthesized ettringite crystals in relation to a saturated solution of calcium hydroxide. The results obtained are presented in Figure 5.

As can be seen from the results obtained in the initial periods of 15 min, 30 min, and 1 h, the concentration of Ca^2+^ ions varied intensively from 22 mmol/L to 16.8 mmol/L and OH^−^ ions from 44 mmol/L to 33.6 mmol/L. Within the next periods of 6, 12, 18 and 24 h, the concentration value changed more smoothly, and after 24 h the concentration of Ca^2+^ ions was 13 mmol/L and OH^−^ 27 mmol/L.

According to the Fajans–Paneth rule [69], only the ions from which the crystal lattice is formed are absorbed: the ions that compose the lattice or isomorphic ions, or the ions that form difficult-to-dissolve compounds with the lattice ions. The ions in the solution in a significant excess will move from the solution to the solid phase and occupy vacant places, completing the crystal lattice. An ettringite structure can adsorb Ca^2+^ ions and OH^−^ ions from solutions. This may be due to the inequality of the electrochemical potentials of the liquid and solid phases.

The ability to absorb certain ions from the solution is determined by the properties of the surface of the additive. One of the characteristics of the interface is the electrokinetic potential (ζ). The value of the ζ-potential was measured by electroosmosis for ettringite 1 h after holding it in distilled water. The value of the ζ-potential was equal to −6 mV. The measured values of the ettringite potential after 1.5 and 3 h after holding it in a solution of calcium hydroxide were 0 mV and +14 mV, respectively. The change of the ζ-potential is explained by the fact that the surface of ettringite crystals, negatively charged when interacting with a saturated solution of calcium hydroxide, is recharged due to the adsorption of Ca^2+^ ions from the solution.

Measurements of the ζ-potential of ettringite obtained by various authors differ significantly from each other. This is due to the different conditions of the studies, different methods of obtaining ettringite and, mainly, the difference in the composition of the solution in contact with crystals [70,71]. The authors of [70] determined that the surface of ettringite crystals in demineralized water is negatively charged, while the value of the ζ-potential was −11.6 mV. When studying the ζ-potential of ettringite in saturated solutions of gypsum and lime, it was shown that its value increases from +8 to +30 mV with an increase in lime concentration in solution [71]. Thus, the measured value of the ζ-potential of ettringite and the comparison of this value with the literature data allowed us to assume that ettringite, like a mineral additive, having a negative surface charge, is able to adsorb positively charged Ca^2+^ ions in saturated solutions of calcium hydroxide. During the adsorption of Ca^2+^ ions, the ettringite surface is recharged, and the value of the ζ-potential increases significantly.

The ability of ettringite crystals to reduce the concentration of Ca^2+^ and OH^−^ ions in a saturated solution of calcium hydroxide, due to the fact that the surface is negatively charged, serves as a basis for the assumption that ettringite crystals can act as a hardening process accelerator in relation to calcium silicates. The authors suggest that, due to this property of ettringite, it can change the rate of dissolution of the silicate component of cement.

### 3.2. Hydration of C_3_S with the Addition of Ettringite

To determine the effect of the addition of ettringite crystals on the hydration of C_3_S, X-ray phase analysis and differential thermal analysis were performed.

**Figure 6:**
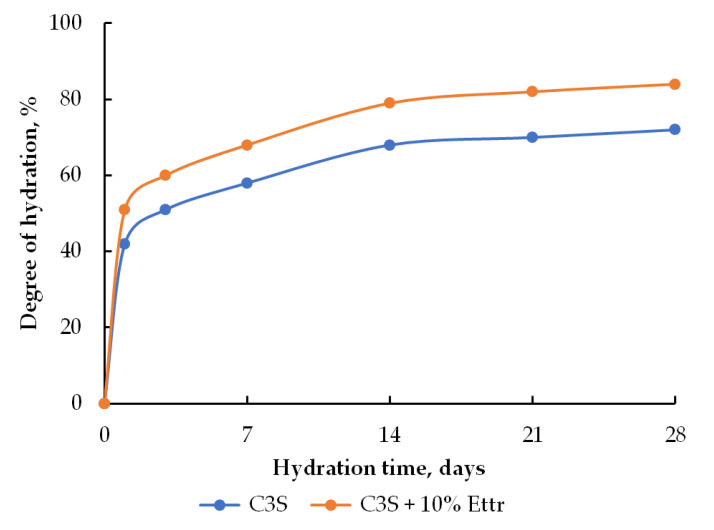
Hydration degree of C_3_S: reference sample and C_3_S modified with 10 wt.% of ettringite.

The C_3_S hydration degree at the 1st and 3rd days of hardening increases in the presence of 10 wt.% of ettringite compared to the reference sample: 51 and 60% for the modified sample and 42 and 49% for the reference sample, respectively (Figure 6).

X-ray phase analysis showed that the composition of the hardened cement stone based on C_3_S and C_3_S with 10 wt.% of ettringite additive differs from the phase composition of the initial mixtures by a decrease in the intensity of peaks at 2θ = 32° corresponding to C_3_S (Figure 1) due to the hydration of the latter, and by the presence of peaks at 2θ = 18° corresponding to calcium hydroxide and a halo corresponding to calcium hydrosilicates.

**Figure 7:**
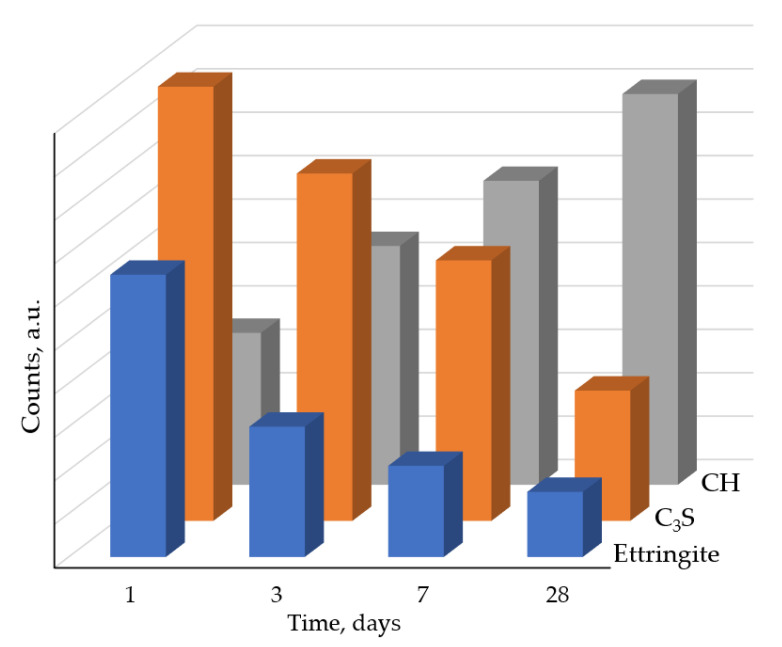
Change in the relative intensity of diffraction peaks of C_3_S sample with 10 wt.% of ettringite during hydration (at 2θ angles): ettringite 9° (Figure 3); C_3_S 32° (Figure 1); calcium hydroxide CH 18°.

Calcium hydrosilicates formed during the hydration of C_3_S are amorphous compounds that do not have clear diffraction peaks on the X–ray patterns, and this complicates their identification using XRD. For C_3_S hydration products, we were able to record only a weak peak at 2θ = 50° and a halo in the region of 2θ = 28–32°, corresponding to calcium hydrosilicates and rather intense peaks corresponding to Ca(OH)_2_. Based on these results, it is possible to assume that the presence of calcium hydrosilicates in the samples does not affect the identification of ettringite or the change in the intensity of ettringite peaks, the analytical peak of which is at 2θ = 9°. As the hydration time increases, the intensity of the diffraction peaks corresponding to calcium hydroxide increases, and the intensity of the diffraction peaks of C_3_S decreases (Figure 7).

To study the dynamics of structural transformations of ettringite crystals during hydration, the profiles of diffraction reflections of the mineral in the area of angles 2θ = 9 and 16° were studied in detail (Figure 3). The results of measurements of profile characteristics are given in Table 3.

The data from Table 3 show that ettringite crystal transformation occurs. An increase in the half-width of the peak at its half-width with a decrease in its intensity indicates the increased diffusivity of ettringite crystals in the presence of calcium hydrosilicates. Such blurring of peaks can be caused by micro-stresses in crystals during their mutual growth with hydrosilicate crystals, which can be seen in SEM microphotographs of hydrated samples.

**Figure 8:**
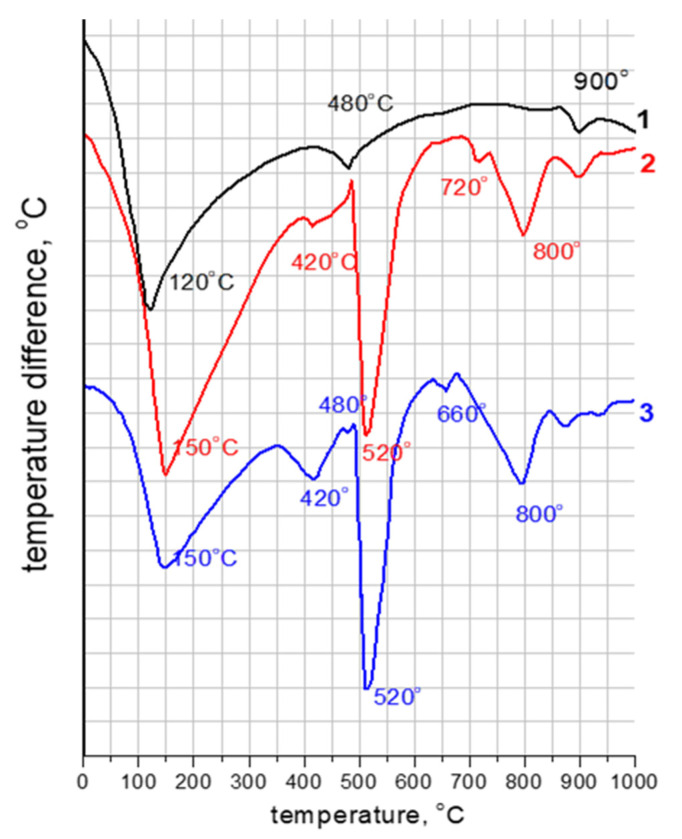
Thermograms of C_3_S hydration products: (1) C_3_S + 10 wt.% of ettringite additive, initial mixture; (2) C_3_S + 10 wt.% of ettringite additive, at 3 days; (3) C_3_S, at 3 days.

The results of the differential thermal analysis are presented in Figure 8 and in Table 4. In the thermograms of the binder from C_3_S with ettringite at 3 days (Figure 8, curve 2) there is a rather large and wide endothermic peak at 150 °C, a blurred endothermic peak at 420 °C, which is a continuation of the first, a very clear and narrow peak at 520 °C, as well as medium-sized peaks at 720 and 800 °C. The endothermic peak at 150 °C is the sum of two effects characterizing the dehydration of ettringite and calcium hydrosilicates. On the thermogram of anhydrousC_3_S with 10 wt.% of ettringite (curve 1), the dehydration of ettringite belongs to the endothermic effect at 120 °C. The endothermic peak at 420 °C is also due to the dehydration of calcium hydrosilicates. A small endothermic peak at 480 °C indicates the removal of water from the highly dispersed Ca(OH)_2_ formed during the decomposition of calcium hydrosilicates. The endothermic peak at 520 °C corresponds to the dehydration of well-crystallized Ca(OH)_2_ formed during the hydration of C_3_S. The endothermic peak at 720 °C is due to the carbonation of calcium hydrosilicates and at 800 °C is due to the decomposition of CaCO_3_, which was formed as a result of the interaction of Ca(OH)_2_ with atmospheric carbon dioxide.

The total mass loss during dehydration of C_3_S samples with ettringite additive is higher than that of samples without an additive and corresponds to 17.8 and 15%, respectively. The amount of Ca(OH)_2_ formed, on the contrary, is higher in samples without the additive than in samples with an ettringite addition and corresponds to 13.6 and 12%, respectively (Table 4). However, if the calculations are made according to the anhydrous calcium silicates, the values are approximately the same: the amount of water in both cases will be 18%, and the amount of calcium hydroxide will be 16%.

Thus, according to the C_3_S degree of hydration and the amount of calcium hydroxide formed, we have shown that the addition of ettringite crystals intensifies the hydration of C_3_S and promotes the formation of calcium hydrosilicates.

### 3.3. Heat Release during Hydration of C_3_S with the Addition of Ettringite

**Figure 9:**
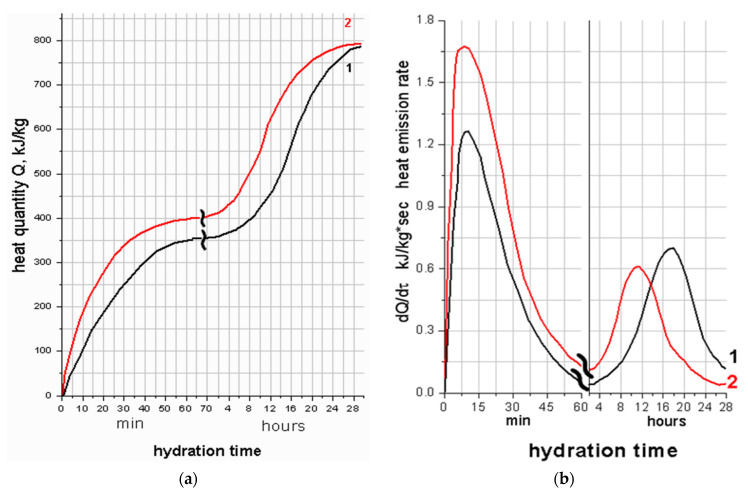
Integral (**a**) and differential (**b**) curves of heat release during hydration: (1) C_3_S; (2) C_3_S + 10 wt.% of ettringite.

Most processes of structure formation and, consequently, mechanical properties of Portland cement are caused by reactions occurring at the initial stages of hydration. The rate of heat release during hydration is directly related to the hydration rate; therefore, we conducted studies on the change of the heat-release rate of C_3_S with ettringite depending on the age of samples. The heat-release curves for C_3_S and C_3_S with ettringite are shown in Figure 9.

The presented heat-release curves have two peaks. The first peak corresponds to the adsorption of water on the active centers and the chemical reaction of the formation of primary hydration products, followed by a slowdown in the hydration process, when the rate of heat release does not change: the so-called induction period. The induction period affects the most important properties of cement paste, its plasticity and workability. The prolongation of the induction stage is associated with a slowdown in the hydration reaction, leading to an extension of the period when the cement paste retains its plasticity and workability and does not harden.

The addition of ettringite increases the intensity of the first peak and slightly reduces the intensity of the second peak. On the differential heat-release curve (Figure 9), the maximum of the second peak shifts towards shorter hydration time. The beginning of the induction period of hydration of non-additive C_3_S and C_3_S with ettringite crystals is the same; in both cases, it occurs after an hour of hydration. The end of the induction period for C_3_S with ettringite occurs much earlier than for that for non-additive C_3_S. An increase in the rate of heat release and the resumption of the hydration reaction of C_3_S hydration occurs after 10 h, and with C_3_S with ettringite after 4.5 h.

Accordingly, the induction period decreases from 11 h for non-additive C_3_S and up to 6 h for C_3_S with 10 wt.% of ettringite. The amount of heat released during 24 h of hydration is higher for C_3_S with ettringite, compared with non-additive C_3_S by 15%. This difference is the maximum from 8 to 16 h and is equal to 30% (Figure 9). After 28 h, the amount of heat released during hydration of C_3_S and C_3_S with ettringite becomes equal.

The microcalorimetric curves of hydration of C_3_S with ettringite are similar to the microcalorimetric curves of hydration of C_3_S with the addition of active silicon dioxide and calcium hydrosilicates [72]. Ettringite additive, similar to amorphous SiO_2_ and calcium hydrosilicates, significantly accelerates the hydration of tricalcium silicate. This is probably due to changes in the concentration of Ca^2+^ ions in the liquid phase, and, consequently, changes in the crystallization conditions of calcium hydrosilicates and Ca(OH)_2_ in the liquid phase and on the surface of additive particles.

### 3.4. Microstructure of Hydrated Samples

During the hydration of C_3_S with 10 wt.% of ettringite, the resulting calcium hydrosilicates precipitate from supersaturated solution onto the surface of ettringite crystals at micro and macro defects. The faster and more intensive the crystallization, the faster the supersaturation decreases, and the formation of stable intergrowth contacts becomes less possible. If we assume that ettringite crystals are not capable of further growth in the medium of hardening C_3_S, the following can be suggested: if ettringite additive particles act as substrates for the nucleation and growth of crystal hydrates in the initial period of hardening, then ettringite crystals further act as a reinforcing filler, since they have the needle-like habit of crystals.

To clarify the processes occurring during the hydration of C_3_S in the presence of ettringite additives, as well as to clarify the question of the stability of ettringite in the medium of hardening C_3_S, the authors conducted electron microscopy studies.

When describing the results, we suggest the following morphological features [73] of calcium hydrosilicates and divide them into four types.

Type I: fibrous particles with l = 0.5–2.0 μm and d = 0.2 μm appear in the first periods (1–28 days) of hardening.

Type II: lamellar particles form, as a rule, a three-dimensional grid of the “honeycomb” type, which appears simultaneously with type I hydrosilicates.

Type III: sections of equal densely packed particles 0.5–0.01 μm can be observed quite rarely in the initial periods of hydration.

Type IV: the so-called “internal product” appears under a shell of type I hydrosilicates.

**Figure 10:**
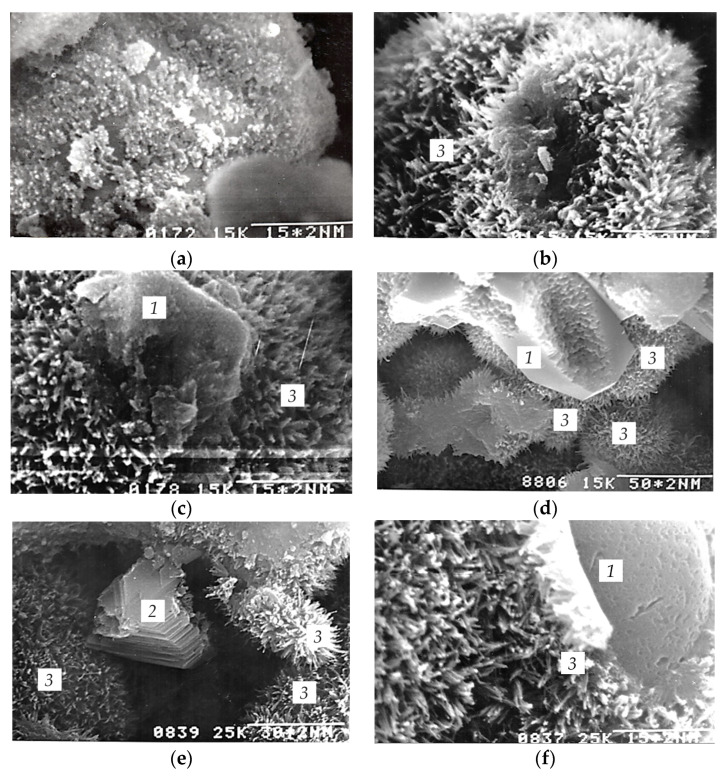
Morphology of C_3_S hydration products: (**a**) 4.5 h of hydration; (**b**) 10 h of hydration; (**c**) 14 h of hydration; (**d**) 1 day of hydration (**e**) 3 days of hydration (**f**) 28 days of hydration; 1: grains of C_3_S, 2: crystals of Ca(OH)_2_, 3: calcium hydrosilicates of morphological type I.

**Figure 11:**
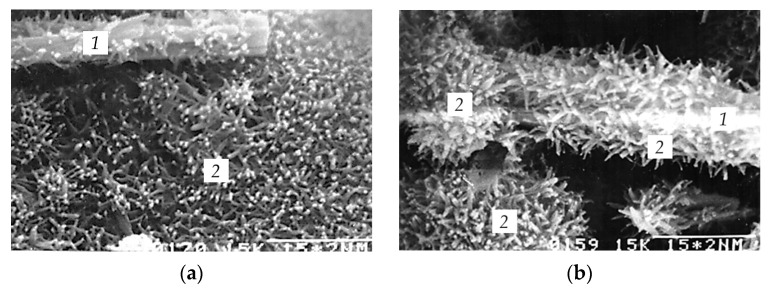

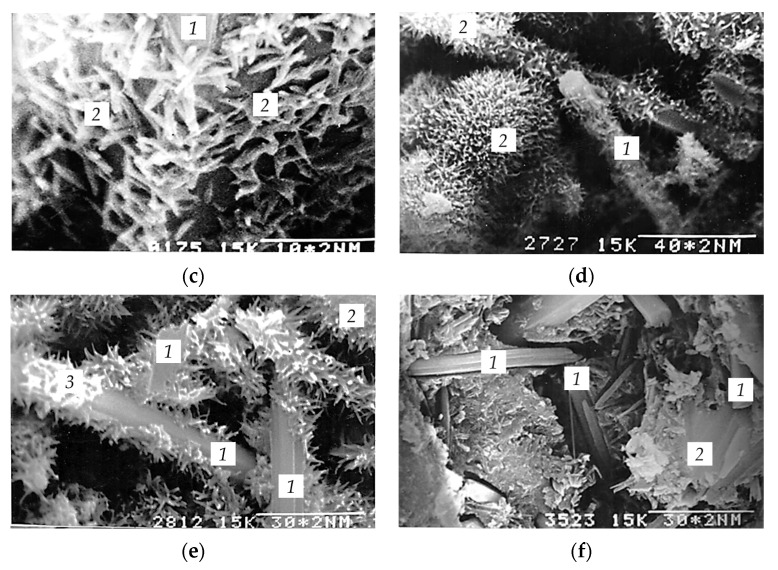
Morphology of C_3_S with 10 wt.% of ettringite hydration products after 4.5 h of hydration: (**a**) 4.5 h of hydration, (**b**) 10 h of hydration, (**c**) 14 h of hydration, (**d**) 1 day of hydration, (**e**) 3 days of hydration, (**f**) 28 days of hydration; 1: ettringite crystals, 2: C_3_S grains coated with calcium hydrosilicates, 3: calcium hydrosilicates of morphological type I.

Samples of 4.5, 10, 14 h of age, 1, 3, 28 days of age and 3 months of age were analyzed. The hardening time of 4.5 h corresponded to the middle of the induction period of non-additive C_3_S and to the end of the induction period of C_3_S with ettringite; that is, the resumption of the hydration process. In 10 h, the end of the induction period of non-additive C_3_S occurs, while for C_3_S with ettringite the second maximum is observed on the heat-release curve. After 14 h of hydration, the second maximum occurs on the differential heat-release curve of non-additive C_3_S, while for C_3_S with ettringite a decrease in the heat-release rate begins. According to standard methods, cement stone samples are tested for compressive and bending strength after 1, 3 and 28 days of hardening. Microphotographs of hydrated samples are shown in Figure 10 and Figure 11.

After 4.5 h of hydration, the non-additive C_3_S samples did not perform strength characteristics and easily crumbled into powder; there was no interaction between calcium hydrosilicates. After 4.5 h, elongated particles formed on the surface of the C_3_S particles with a size of 50–70 nm, obviously the calcium hydrosilicate particles (Figure 10a).

After 10 h of hydration, the non-additive C_3_S samples already had sufficient strength; the hydration products were represented by morphological type I calcium hydrosilicates with a size of 0.4–0.6 μm (Figure 10b). The destruction of the sample occurred through a porous hydrosilicate at locations of the weakest adhesive contacts.

After 14 h of hydration, the same situation was observed, while the size of the hydrosilicates did not change (Figure 10c). In the sample at 1 day, hydration products were mainly represented by morphological type I calcium hydrosilicates; the formation of Ca(OH)_2_ crystals with a size of 1–2 μm was observed, and the presence of individual C_3_S grains with a size of 5–7 μm of a regular shape with selective dissolution in the center of the grain could also be noted (Figure 10d). On the fracture surface there are C_3_S particles coated with morphological type I calcium hydrosilicates, as well as calcium hydrosilicates crystallizing in the interstitial space in places of high local supersaturation and having a globular shape.

After 3 days of hardening, morphological type I calcium hydrosilicate becomes the predominant hydration product, while the particle size increased slightly up to 0.5–0.7 μm (Figure 10e). Locally, there are packages of calcium hydroxide crystals of 3–4 μm. Individual areas of Ca(OH)_2_ can be observed at a close distance from calcium hydrosilicates formed in the process of dissolution or crystallization. On the contrary, the areas at a further distance from calcium hydrosilicates are clear-shaped. This indicates that equilibrium has not yet been reached in the system. The structure of the solidified sample remains quite porous and loose. The destruction of the sample occurs mainly through the areas of contact between hydrosilicate layers formed near the surface of the initial C_3_S grains. In this period, the interaction between individual calcium hydrosilicates increases and exceeds the adhesive interaction between calcium hydrosilicates and C_3_S particles, as well as the energy of destruction of the C_3_S grains. Therefore, in some places, the destruction of the sample is accompanied by the separation of the hydrosilicate layer from the initial C_3_S particles and the destruction of C_3_S grains. Anhydrous C_3_S grains can be observed on the fracture surface.

After 28 days of hardening, the structure is compacted, and individual pores are covered with hydration products (Figure 10f). Hydrosilicate products are represented by hydrosilicates of morphological type I and IV. The size of calcium hydrosilicates of morphological type I was 1–1.5 μm, and the size of the calcium hydrosilicate layer of type IV reached 5–7 μm. There are packages of calcium hydroxide crystals between calcium hydrosilicates that have a very clear shape; the size of the calcium hydroxide crystals is 5–7 μm, and the thickness of individual crystals is 0.5 μm. There are also anhydrous grains of C_3_S of 5–10 μm in size, surrounded by a layer of calcium hydrosilicates of the morphological types IV and I. In the intervals between the hydrated grains, hydrosilicates of morphological type I of globular shape are located. The energy of interaction between hydration products exceeds the energy of destruction of hydrated C_3_S grains; the crack surface becomes quite smooth, and there are hydrated C_3_S grains in close contact and calcium hydroxide crystals formed between them. Locally, the formation of cracks is observed; the cracks pass through the contact points of individually hydrated grains, the hydrosilicate formations and cleavage planes of Ca(OH)_2_.

C_3_S with 10 wt.% of ettringite samples after 4.5 h of hydration already have sufficient strength, unlike non-additive C_3_S samples, and do not crush when being prepared for analysis. A significant amount of calcium hydrosilicates is formed on the surface of C_3_S particles and ettringite crystals, which can be attributed to morphological type I (Figure 11a). The size of calcium hydrosilicates is 0.1–0.2 μm. After 10.5 and 14 h of hydration, the amount of hydrosilicates and their size continue to increase (Figure 11b,c), from 0.2–0.3 μm and 0.3–0.4 μm, respectively. The interaction between individual calcium hydrosilicates is still very insignificant in this period; therefore, destruction occurs through a hydrosilicate shell.

In C_3_S with 10 wt.% of ettringite samples at 1, 3 and 28 days, morphological type I hydrosilicates continue to crystallize on the lateral faces of ettringite crystals, similar to those that appear on the surface of non-additive C_3_S.

Calcium hydrosilicates are elongated and point-shaped crystals. The crystal axis is oriented radially to the ettringite crystal axis. With increasing hydration time, the hydrosilicates formed on the surface of ettringite crystals do not disappear, but continue to grow. Their size at 1, 3 and 28 days is 0.4; 0.8; 1.5 μm, respectively (Figure 11d–f).

Obviously, the concentrations of the corresponding ions in the liquid phase are sufficient for the growth of calcium hydrosilicates. The density of precipitated hydrosilicates is higher on ettringite crystals that are located near C_3_S grains. The closer the dissolving compound to the crystallization center, the faster the ions reach the crystallization surface where local supersaturation for the crystallization of a new hydrate phase is reached. The ettringite crystals furthest from C_3_S particles remain smooth, without noticeable changes. It is clearly seen that ettringite crystals loosen the structure and prevent the formation of a dense structure by the hydration products, and the number of adhesive and intergrowth contacts is reduced. This is the reason the positive effect of ettringite particles as crystallization centers does not lead to a noticeable increase in the strength of the samples.

At the first day, the destruction of the system occurs along the hydrosilicate area, while on the fracture surface individual C_3_S particles can be observed coated with hydrosilicate gel-like shells, globular hydrosilicate particles, and ettringite crystals coated with hydration products. At 3 days, the interaction between individual hydrosilicates increases and the destruction of the system occurs not only through the porous products, but also through the C_3_S particles, cleavage planes of calcium hydroxide, as well as through areas of the weakest adhesive interaction of ettringite crystals with calcium hydrosilicates.

### 3.5. Kinetics of the Solid-Phase Nucleation during the Setting of C_3_S Samples

**Figure 12:**
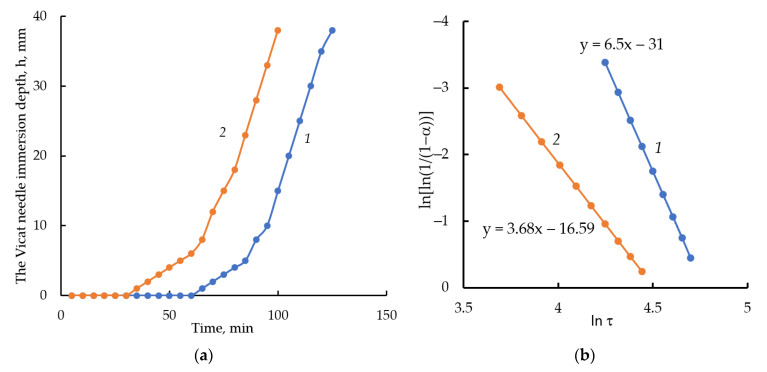
The kinetics of setting (**a**) of the paste samples with normal consistency C_3_S (1) and C_3_S with 10% ettringite (2) according to measurements with the Vicat apparatus; (**b**) areas of increasing sample density in the coordinates of Avrami–Erofeyev Equation (5) for samples C_3_S (1) and C_3_S with the addition of 10% ettringite (2).

The kinetics of setting of the C_3_S and C_3_S with 10 wt.% of ettringite pastes of standard consistency are shown in Figure 12. The results show that the setting time of C_3_S with 10 wt.% of ettringite is reduced both at the beginning of structure formation and at the end of the hardening process. This is due to the fact that the addition of ettringite crystals provides more nucleation centers and reduces the energetic barrier required during the nucleation process. The value of the indicator *n* represents the dynamics of new-phase growth according to the Avrami–Erofeyev model [67]. For the control sample C_3_S, the value of parameter *n* is 6.5 (*n* > 4); for the sample C_3_S modified with ettringite, this value is significantly less and equals 3.68 (*n* < 4) (Figure 12). In accordance with the nucleation model [67], the setting and hardening of cement paste due to the crystallization of hydration products in the case of a sample modified with ettringite proceeds in the presence of already formed nucleation centers of crystalline phases, which reduces the energy barrier of this process and increases the rate of formation of the cement stone structure.

### 3.6. Strength of Hydrated C_3_S Samples

The bending and compressive strength of C_3_S samples with ettringite additive at the first day of aging was approximately 30% lower than that of non-additive samples (Table 5). At 3 days, the strength values of the reference samples with the additive were almost the same. At 28 days, the bending strength of the samples with the additive was 10% higher than the bending strength of non-additive samples, while the compressive strength was 20% less.

An increase in bending strength (by the third day of hardening and further) greater than the increase in compressive strength in samples of C_3_S with ettringite indicates that needle crystals of ettringite act as a reinforcing filler.

Thus, it is shown in the work that the introduction of ettringite crystalline additive is a useful way to study the mechanisms of early hydration of cement minerals. The accelerating properties of microcrystalline ettringite observed in the work depend on the nucleation and growth of hydrosilicates forming at the early stage of the hydration process. An increase in the hydration activity of C_3_S in the presence of an ettringite additive, observed by a change in the dynamics of the degree of hydration, heat release, structure formation and growth of nuclei of crystalline phases, indicates the prospects for the practical application of such an additive to cements. Acceleration of structure formation in cement materials is fundamentally important for innovative construction technologies, in particular, for 3D printing of building products and structures.

## 4. Conclusions

The kinetics of hydration of C_3_S with 10 wt.% of ettringite was studied for the first time. It was found that the addition of ettringite significantly accelerates the crystallization of calcium hydrosilicates but does not change the morphology of hydration products in general. The acceleration of calcium hydrosilicate crystallization leads to a decrease in the induction period on the heat-release curve. The induction period of C_3_S with 10 wt.% of ettringite is reduced by half. The degree of hydration of C_3_S with 10 wt.% of ettringite sample at 1 day increased by 10% compared to the non-additive C_3_S sample. Significant amounts of calcium hydrosilicates are formed on the surface of ettringite crystals located near hydrating C_3_S grains. Their size increases from 0.2 μm (after 10 h of hydration) to 1.5 μm (after 23 days of hydration).

It has been established that ettringite crystals have a negative electrokinetic potential and are capable of adsorbing Ca^2+^ ions from saturated solutions of calcium hydroxide. Ettringite crystals can act as a hardening accelerator additive in relation to calcium silicates. Due to this, ettringite can change the rate of dissolution of the silicate component of cement.

The addition of ettringite crystals to Portland cement reduces the time required for the beginning of the nucleation process, which leads to a faster hydration rate of cement minerals. Needle-shaped ettringite crystals can act as a reinforcing filler.

## Figures and Tables

**Table 1:** Chemical composition of C_3_S (wt.%).

SiO_2_	CaO	Al_2_O_3_	Fe_2_O_3_	Other Oxides
26.35 ± 0.03	73.24 ± 0.05	0.10 ± 0.01	0.03 ± 0.002	0.28 ± 0.05

**Table 2:** Ratios for calculating the amount of dry residue from calcium silicates.

Parameter	C_3_A∙3CaSO_4_∙32H_2_O Additive
Amount of dry residue from hydrated and non-hydrated C_3_S in the mixture	$\frac{100-m}{100\cdot 228}$
Amount of solid residue introduced into the system with the additive	$\frac{m}{100\cdot 678}$
Amount of water introduced into the system with the additive	$\frac{m}{100\cdot 540}$

**Table 3:** Dynamics of changes in the characteristics of ettringite diffraction reflection profiles in hydrated C_3_S samples with 10 wt.% of ettringite according to XRD data.

No.	Sample	Peak Parameter (at 2θ Angle)							
		9°				16°			
		d, nm	I, %	Half-Width	Area	d, nm	I, %	Half-Width	Area
1	Initial	0.9782	95	0.20	18.9	0.563	57	0.20	13
2	1 day	0.979	88	0.22	19.3	5.609	33	0.21	7.04
3	3 days	0.9805	63	0.24	15.5	5.644	25	0.21	5.21
4	28 days	0.9814	19	0.36	6.81	5.630	8	0.23	1.9

**Table 4:** DTA results for C_3_S samples with ettringite additive.

Sample	Sample Mass, G, mg	Total Mass Loss		Anhydrous CS, mg	Total Ca(OH)_2_, mg	Hydrate Water, wt. %	Increase in Ca(OH)_2_, wt. %
		$∆G^{20-100},$ mg	$∆G^{100-1000},$ mg				
C_3_S + 10 wt.% of ettringite, initial mixture	1000	46	10	900	-	-	-
C_3_S, at 3 days	1000	150	130	850	136	18.0	16.0
C_3_S + 10 wt.% of ettringite, at 3 days	1000	178	155	775	120	18.0	16.0

**Table 5:** Strength (MPa) of C_3_S samples.

Amount of Ettringite Additive, wt.%	Standard Consistency W/C	Hydration Time					
		1 Day		3 Days		28 Days	
		Bending Strength	Compressive Strength	Bending Strength	Compressive Strength	Bending Strength	Compressive Strength
−	0.42	3.6 ± 0.2	12.0 ± 0.5	6.0 ± 0.3	22.0 ± 0.8	12.0 ± 0.5	87.0 ± 1.1
10	0.45	2.5 ± 0.3	9.0 ± 0.5	6.8 ± 0.2	22.0 ± 0.9	13.0 ± 0.4	71.0 ± 1.2

## Data Availability

The data presented in this study are available on request from the corresponding author.
